# In Memoriam—L. S. Reynolds, M. D.

**Published:** 1890-03

**Authors:** 


### IN MEMORIAM—L. S. REYNOLDS, M. D.

Again we have to record the death of an earnest and faithful brother in medicine, a true homœopathic student and homœopathic practitioner, Dr. L. S. Reynolds.

Among the earliest students of Homœopathy he followed in the footsteps of his very dear friend, Dr. Noah Harmer, of Buffalo, the pioneer of Homœopathy in Western Pennsylvania, and was also closely united in esteem and friendship with Dr. P. P. Wells, of New York, and with them his faith in the true old practice of Hahnemannian theory and principles was loyal and intense. The youngest man of the three, his remarkable intelligence and information on all scientific subjects, particularly medicine, caused him to be especially appreciated by men of superior intellect.

He was born in New York seventy-six years ago. The early years of his manhood were spent in Buffalo, N. Y.

For thirty years past his home has been in Brooklyn, N. Y. Since his advance in years he has confined his practice mostly to suffering patients he attended gratuitously, as his greatest pleasure in life was to relieve suffering; permitted so to do through instinctive and wonderful knowledge of materia medica. Those who have lost him best know his worth, and Homœopathy in its pure perfectness has truly lost a faithful believer and disciple.

